# An Improved Circular Fringe Fourier Transform Profilometry

**DOI:** 10.3390/s22166048

**Published:** 2022-08-12

**Authors:** Qili Chen, Mengqi Han, Ye Wang, Wenjing Chen

**Affiliations:** Department of Opto-Electronics, Sichuan University, Chengdu 610065, China

**Keywords:** 3D surface measurement, circular fringe projection, co-ordinate transformation, Fourier transform profilometry

## Abstract

Circular fringe projection profilometry (CFPP), as a branch of carrier fringe projection profilometry, has attracted research interest in recent years. Circular fringe Fourier transform profilometry (CFFTP) has been used to measure out-of-plane objects quickly because the absolute phase can be obtained by employing fewer fringes. However, the existing CFFTP method needs to solve a quadratic equation to calculate the pixel displacement amount related to the height of the object, in which the root-seeking process may get into trouble due to the phase error and the non-uniform period of reference fringe. In this paper, an improved CFFTP method based on a non-telecentric model is presented. The calculation of displacement amount is performed by solving a linear equation instead of a quadratic equation after introducing an extra projection of circular fringe with circular center translation. In addition, Gerchberg iteration is employed to eliminate phase error of the region close to the circular center, and the plane calibration technique is used to eliminate system error by establishing a displacement-to-height look-up table. The mathematical model and theoretical analysis are presented. Simulations and experiments have demonstrated the effectiveness of the proposed method.

## 1. Introduction

Fringe projection profilometry (FPP), as a common active optical three-dimensional (3D) measurement technology, has advantages of high-precision, non-contact, and full-field measurement [1,2,3,4]. In a fringe projection system based on a triangular configuration frame, the structured fringe patterns are projected onto the object by a projector, then the distorted images will be captured by a camera from another view angle. The height of the measured object will change the phase distribution of the fringe, which can be obtained by different demodulation algorithms based on the number of fringes. The straight fringe and oblique fringe are popular [5,6]. Saw-tooth fringe [7], triangular fringe [8], hexagonal fringe [9], circular fringe [10,11], etc. have been used in fringe projection profilometry as well. To reconstruct phase information from these patterns, algorithms based on phase shifting [2], Fourier transform [3,12,13,14,15], wavelet transform [16,17], or windowed Fourier transform have been developed [18]. The comparative analysis of different carrier fringe pattern techniques has been given in references [19,20].

Fourier fringe analysis is one of the most popular methods aimed at calculating the phase value from a single spatial carrier pattern or at most two spatial carrier patterns through Fourier transform, filtering operation and inverse Fourier transform. In traditional Fourier transform profilometry (FTP), the popularly projected fringe pattern is a sinusoidal straight or oblique fringe for the ease of extraction of the fundamental spectrum lobe carrying surface information of the measured object. However, one disadvantage of linear fringe projection is that the unwrapped phase map obtained by spatial unwrapping algorithms [21,22] has 2π ambiguity because the value of the continuous phase depends on the unwrapping starting point. For eliminating this ambiguity, a common method is embedding a marker point into fringe patterns or adding a marker on the surface of the object. The marker provides a reference for unwrapping the phase. Certainly, temporal phase unwrapping methods such as multi-frequency and multi-wavelength approaches [23,24] can be used to obtain the absolute phase by determining 2π discontinuous locations. However, a series of fringe patterns with different frequencies are needed.

Circular fringe projection profilometry (CFPP) has also attracted research interest in recent years [10,11,25,26] because the center of the circular fringe pattern provides a reference mark. Ratnam et al. [25] measured the out-of-plane deformation of targets by calculating the pixel displacement of the circular fringe pattern. Mandapalli et al. [26] used the circular fringe projection method to measure the 3D profiling of high dynamic range objects. Zhao et al. [27] achieved the 3D profile measurement via the triangular structure between a projected divergent light ray and the optical axis of the projector in a coaxial system. Wang et al. [28] used circular gratings to perform moiré-based misalignment measurements combined with lithography. In addition, the conical phase image has other applications. For example, Khonina et al. [29] analyzed the wavefront aberrations of the interferograms using a conical reference beam and neural networks processing.

Circular fringe Fourier transform profilometry (CFFTP) based on a triangulation system has the capability of whole out-of-plane measurement using fewer fringes. To the best of our knowledge, the existing CFFTP [25,26] calculates the displacement amount carrying the height information of the object by solving a quadratic equation. The theoretical model is applicable to the telecentric system. The correct root-seeking process of the quadratic equation may get into trouble due to the phase error and the non-uniform period of the reference fringe. Thus, interpolation and fitting are required to deal with the error region in the middle of the image. In addition, phase error near the center region is bigger because of the leakage of the spectrum.

In this paper, some improvements are presented for the generality and accuracy of CFFTP. To avoid the trouble of root-seeking, the expression of calculating the displacement amount is degraded to a linear equation from a quadratic equation by introducing an extra projected circular fringe with a circular center lateral shift. Compared to the existing CFFTP, the theoretical model of our method is also suitable for a system whose projection and imaging centers are at a finite distance. In addition, Gerchberg iteration is employed to eliminate error close to the circular center region, and an established look-up table describing the relationship between displacement and height is used to eliminate system error of the CFFTP. Results of simulations and experiments illustrate that our improved CFFTP offers the capability of measuring out-of-plane deformation with higher accuracy and robustness.

The rest of this paper is organized as follows. Section 2 describes the principles of the improved CFFTP, including mathematical model and displacement amount calculation, co-ordinate transformation, conical phase calculation by FTP, and the displacement-to-height look-up table. Section 3 and Section 4, respectively, present some simulations and experiments to validate the proposed method. Section 5 summarizes the paper.

## 2. Principle

### 2.1. Geometric Model and Calculation of Lateral Displacement of CFFTP

**Figure 1:**
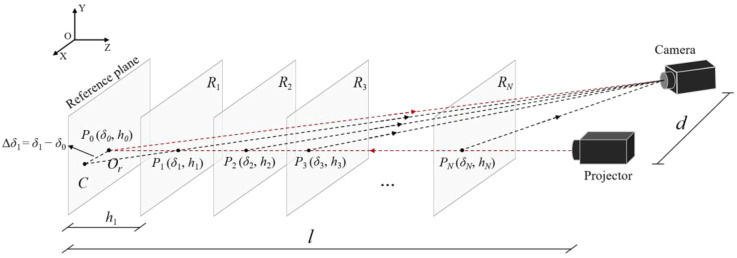
Schematic diagram of the measurement system.

The schematic diagram of the geometric model of CFFTP is the same as that of traditional FPP based on the triangulation principle, as shown in Figure 1. On the top-left of this figure, an orthogonal co-ordinate system is determined for exhibiting spatial directions, where *d* and *l* are structural parameters of the measurement system. The optical axis of the projector and the camera intersects the point $O_{r}$ on the reference plane. The plane in which two optical axes lie is parallel to the *X*–*Z* plane. Several planes $R_{i}$(*i* = 1, 2,…, *N*) are drawn to exhibit out-of-plane height. The lateral shift of the fringe pattern caused by the measured object will be along the *X*-axis direction when a circular pattern is projected onto the object and captured by the camera. This lateral shift is related to the phase information, which is thereby used to restore the surface of the object. For clarity, taking an emitting ray from a pixel of the projector as an example, the intersections of the ray and each plane are $P_{i}$(*i* = 0, 1,…, *N*), which have the same phases. These points are captured by different pixels on the camera. $P_{i}$ relates to a set of data pairs ($\delta_{i}$, $h_{i}$) which means that different heights $h_{i}$ correspond to different lateral shift $\delta_{i}$. The lateral shift can be obtained by calculating the phase difference of homologous points. For instance, point $P_{1}$ on the plane *R* and point *C* on the reference plane are “seen” by the same pixel on the camera, but they have different encoding phase values. The phase difference between $P_{1}$ and *C* describes the displacement amount between *P*_0_ and *C*, which is used to calculate the lateral shift amount Δ$\delta_{1}$. That is, Δ$\delta_{1}$ = $\delta_{1}-\delta_{0}$ denotes the lateral shift caused by height $h_{1}$.

To accurately calculate the lateral shift amount, an improved CFFTP method is proposed. Two circular fringes with the same period and different circular centers (*k* pixels lateral shift in the *x* direction) are generated. The encoded circular fringes can be expressed as:

(1) $$I_{1}\left(x_{p},y_{p}\right)=a+b\cos(\frac{2\pi \sqrt{{\left(x_{p}-x_{p0}\right)}^{2}+{\left(y_{p}-y_{p0}\right)}^{2}}}{p}),$$

(2) $$I_{2}\left(x_{p},y_{p}\right)=a+b\cos(\frac{2\pi \sqrt{{\left(x_{p}-x_{p0}+k\right)}^{2}+{\left(y_{p}-y_{p0}\right)}^{2}}}{p}),$$

where (*x*_*p*_, $y_{p}$) is the pixel co-ordinate of an LCD/DLP plane, *p* is the encoded fringe period, ($x_{p0}$, $y_{p0}$) and ($x_{p0}$ − *k*, $y_{p0}$) are the centers of two circular fringes respectively. They are projected on the reference plane and the measured object. In the non-telecentric measurement system, the reference fringes and the deformed fringes captured by the camera can be expressed as:

(3) $$I_{c1}\left(x_{c},y_{c}\right)=a_{c}(x_{c},y_{c})+b_{c}(x_{c},y_{c})\cos(\frac{2\pi \sqrt{{\left(x_{c}-x_{c0}+\delta_{x0}\right)}^{2}+{\left(y_{c}-y_{c0}\right)}^{2}}}{p_{c}}),$$

(4) $$I_{c2}\left(x_{c},y_{c}\right)=a_{c}\left(x_{c},y_{c}\right)+b_{c}\left(x_{c},y_{c}\right)\cos(\frac{2\pi \sqrt{{\left(x_{c}-x_{c0}+\delta_{x0}+k_{c}\right)}^{2}+{\left(y_{c}-y_{c0}\right)}^{2}}}{p_{c}}),$$

(5) $$I_{c3}\left(x_{c},y_{c}\right)=a_{c}\left(x_{c},y_{c}\right)+b_{c}\left(x_{c},y_{c}\right)\cos(\frac{2\pi \sqrt{{\left(x_{c}-x_{c0}+\delta_{x}\right)}^{2}+{\left(y_{c}-y_{c0}\right)}^{2}}}{p_{c}}),$$

(6) $$I_{c4}\left(x_{c},y_{c}\right)=a_{c}\left(x_{c},y_{c}\right)+b_{c}\left(x_{c},y_{c}\right)\cos(\frac{2\pi \sqrt{{\left(x_{c}-x_{c0}+\delta_{x}+k_{c}\right)}^{2}+{\left(y_{c}-y_{c0}\right)}^{2}}}{p_{c}}),$$

where subscript *c* indicates the camera, and ($x_{c}$, $y_{c}$) is the camera pixel co-ordinate; $\delta_{x0}$ is the original lateral shift amount of the reference fringe caused by the triangular relationship of the measurement system, while $\delta_{x0}$ equals zero in the telecentric measurement system; $\delta_{x}$ is the lateral shift amount in the deformed fringe; $k_{c}$ is the offset of the two circular centers of fringes; Hence, Δ$\delta_{x}$ = $\delta_{x}$ − $\delta_{x0}$ is the lateral shift amount caused by the measured object, which is also a function of pixel co-ordinate ($x_{c}$, $y_{c}$). The terms in brackets of the cosine functions in Equations (3)–(6) are the phases of the fringes, which can be abbreviated as $\varphi_{1}$, $\varphi_{2}$, $\varphi_{3}$, and $\varphi_{4}$ respectively. If these phases are extracted (see Section 2.3), Δ*δ_x_* can be calculated by solving a linear equation in our work instead of a quadratic equation in the existing CFFTP. A simple deduction is given in the following.

Calculating the difference of the square of the phase terms of Equations (3) and (5), we obtain the following equation:

(7) $$\delta_{x}^{2}-\delta_{x0}^{2}+2(x_{c}-x_{c0})(\delta_{x}-\delta_{x0})={\left(\frac{p_{c}}{2\pi }\right)}^{2}(\varphi_{3}^{2}-\varphi_{1}^{2}).$$

Similarly, from Equations (4) and (6), we obtain:

(8) $$\delta_{x}^{2}-\delta_{x0}^{2}+2(x_{c}-x_{c0}+k_{c})(\delta_{x}-\delta_{x0})={\left(\frac{p_{c}}{2\pi }\right)}^{2}(\varphi_{4}^{2}-\varphi_{2}^{2}).$$

From Equations (7) and (8), the lateral displacement caused by the height of the measured object is expressed as:

(9) $$\Delta \delta_{x}=\delta_{x}-\delta_{x0}=\frac{p_{c}^{2}}{8k_{c}\pi^{2}}(\varphi_{4}^{2}-\varphi_{2}^{2}-\varphi_{3}^{2}+\varphi_{1}^{2}).$$

Equation (9) is more general compared with the formula of displacement in the existing CFFTP, like Equation (7). The value of Δ$\delta_{x}$ cannot be calculated by simply solving the quadratic equation because $\delta_{x0}$ is unknown in the non-telecentric system. Even if $\delta_{x0}$ is ignored, seeking the correct root is not easy because solving the quadratic equation may become inaccurate around the middle region. Therefore, interpolation and fitting operations are required in the existing CFFTP. The proposed linear equation can well avoid this problem.

### 2.2. Coordinate Transformation of CFFTP

As Fourier transform profilometry cannot directly process closed fringes, co-ordinate transformation from a Cartesian to Polar co-ordinate must be performed in CFFTP. The resulting fringes in Polar co-ordinate are expressed as:

(10) $$I_{n}^{\prime }(r_{n},\theta_{n})=a^{\prime }(r_{n},\theta_{n})+b^{\prime }(r_{n},\theta_{n})\cos(\frac{2\pi r_{n}}{p\prime }),\;\;\;\;\;\;\;\;\;n=1,2,3,4,$$

where $r_{n}$, $\theta_{n}$, and *p*’ are the radial variables, the angle variables, and the period of fringe in a Polar co-ordinate respectively. *a’*($r_{n}$, $\theta_{n}$) and *b’*($r_{n}$, $\theta_{n}$) are the background intensity and modulation intensity. The circular centers correspond to the origin of the Polar co-ordinate images. The range of $\theta_{n}$ is [0, 360°), $r_{n}$ and $\theta_{n}$ are shown in Equations (11)–(14).

(11) $$r_{1}=\sqrt{{\left(x_{c}-x_{c0}+\delta_{x0}\right)}^{2}+{\left(y_{c}-y_{c0}\right)}^{2}},\theta_{1}=\arctan(\frac{y_{c}-y_{c0}}{x_{c}-x_{c0}+\delta_{x0}}),$$

(12) $$r_{2}=\sqrt{{\left(x_{c}-x_{c0}+\delta_{x0}+k_{c}\right)}^{2}+{\left(y_{c}-y_{c0}\right)}^{2}},\theta_{2}=\arctan(\frac{y_{c}-y_{c0}}{x_{c}-x_{c0}+\delta_{x0}+k_{c}}),$$

(13) $$r_{3}=\sqrt{{\left(x_{c}-x_{c0}+\delta_{x}\right)}^{2}+{\left(y_{c}-y_{c0}\right)}^{2}},\theta_{3}=\arctan(\frac{y_{c}-y_{c0}}{x_{c}-x_{c0}+\delta_{x}}),$$

(14) $$r_{4}=\sqrt{{\left(x_{c}-x_{c0}+\delta_{x}+k_{c}\right)}^{2}+{\left(y_{c}-y_{c0}\right)}^{2}},\theta_{4}=\arctan(\frac{y_{c}-y_{c0}}{x_{c}-x_{c0}+\delta_{x}+k_{c}}).$$

**Figure 2:**
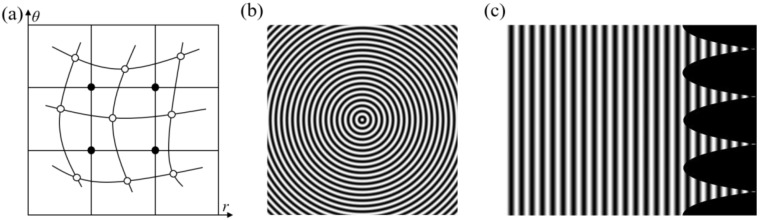
Diagram of co-ordinate transformation. (**a**) Interpolation schematic diagram; (**b**) Original circular fringe; (**c**) Linear fringe in Polar co-ordinate.

To obtain fringes in a Polar co-ordinate, a gridded sampling operation has to be worked on $r_{n}$ and $\theta_{n}$. Those are expressed as $r_{n}$(*i*, *j*) and $\theta_{n}$(*i*, *j*), *i* = 1, 2, …, $K_{1}$, *j* = 1, 2, …, *K*_2_. The resolution of polar images is $K_{1}\times K_{2}$. The denser the sampling points are, the less the error caused by the co-ordinate transformation operation. But an over high-resolution ratio costs more computing time. In this paper, $K_{1}$ is obtained by sampling *θ*(*i*, *j*) by 0.25 degrees as an interval, and $K_{2}$ is obtained by sampling $r_{n}$(*i*, *j*) by 0.5 pixels as an interval. In practice, each pixel of circular fringes may not fall on the corresponding gridded point after being transferred to the Polar co-ordinate, so interpolation calculation is necessary. Figure 2 shows a schematic diagram of co-ordinate transformation. Figure 2a shows the interpolation procedure. For example, white points denote corresponding polar points calculated by Equation (11) directly, and black points are the gridded points. The intensity of each white point is equal to that of the corresponding point in the circular fringe. The intensity of each black point is calculated by an interpolation operation employing its neighborhood white points. There are many interpolation algorithms [30,31,32]. The cubic interpolation algorithm is used in our simulations and experiments. Figure 2b is a reference circular fringe and Figure 2c is its corresponding fringe in a Polar co-ordinate, which can be processed by Fourier transform profilometry.

### 2.3. Calculation of Conical Phase by FTP

To calculate phases, Fourier transform, filtering operation, and inverse Fourier transform are performed to deal with the fringes described by Equation (10). The Fourier spectra of the four fringes are expressed as:

(15) $$F\{I_{n}^{\prime }(r_{n},\theta_{n})\}=A_{n}(f_{r_{n}},f_{\theta_{n}})+\frac{1}{2}B_{n}(f_{r_{n}}-f_{0_{n}},f_{\theta_{n}})+\frac{1}{2}B_{n}^{*}(f_{r_{n}}+f_{0_{n}},f_{\theta_{n}}),\;\;\;\;\;\;\;\;\;n=1,2,3,4,$$

where * denotes complex conjugate. *f_rn_* and *f_θn_* are the variables in the frequency domain, *f*_0*n*_ is the carrier frequency of each linear fringe. $A_{n}$(*f_rn_*, *f_θn_*) is zero frequency component, $B_{n}$(*f_rn_* − *f*_0*n*_, *f_θn_*) and $B_{n}^{*}$(*f_rn_* + *f*_0*n*_, *f_θn_*) are fundamental frequency components. One of the fundamental frequency components can be selected by applying a band-pass filter and the inverse Fourier transform is performed on it to calculate the wrapped phase ranging from −π to π with 2π modus. Thus, a suitable spatial phase unwrapping algorithm is used to obtain the absolute phase by selecting a point within the first linear pitch as the unwrapping starting point. As mentioned above, the obtained four conical phases $\varphi_{1}$, *φ*_2_, $\varphi_{3}$, and $\varphi_{4}$ are used to calculate the lateral displacement.

It is worth noting that the circular center of each circular fringe in the Cartesian co-ordinate corresponds to the left edge of the resulting images in the Polar co-ordinate. When Fourier transform works on these fringes directly, phase accuracy in the edge regions is influenced by spectral leakage. After the phases are converted back to the Cartesian co-ordinate, the corresponding areas of the conical phase maps have bigger errors. To solve this problem, an extrapolation operation is required to extend the boundary of fringes. The Gerchberg algorithm [33] is an effective solution to eliminate edge leakage error. We will use it to reduce phase error in our simulations and experiments.

### 2.4. Establishment of the Displacement-to-Height Mapping

According to the geometric model of the measurement system shown in Figure 1, theoretically, the relationship between displacement Δ*δ*($x_{c}$, $y_{c}$) and height *h*($x_{c}$, $y_{c}$) can be expressed as:

(16) $$h(x_{c},y_{c})=\frac{\Delta \delta_{x}(x_{c},y_{c})l}{\Delta \delta_{x}(x_{c},y_{c})+d}.$$

Since the system parameters, *d* and *l* are difficult to measure accurately, and fringe period $p_{c}$ of the reference fringes is not a constant for a non-telecentric system, it is necessary to establish a look-up table between Δ$\delta_{x}$($x_{c}$, $y_{c}$) and *h*($x_{c}$, $y_{c}$) by the plane calibration method [34]. To set up the relationship, the reference plane is moved along the *Z*-axis, and fringes at known positions $h_{i}$ (on plane $R_{1}$ to $R_{N}$) are captured by the camera. At each position, the phases of the fringes are calculated by our method. The phase differences are used to calculate lateral displacement Δ$\delta_{x}$. Linear fitting method, quadratic fitting method, cubic fitting method, or higher-order polynomial fitting can be used to set up a look-up table according to the number of calibration planes [35]. Considering the balance between time-consumption and accuracy, the cubic polynomial fitting method is selected to set up the look-up table in our experiment, which can be expressed as:

(17) $$\begin{array}{ll} h(x_{c},y_{c})= & a_{0}(x_{c},y_{c})+a_{1}(x_{c},y_{c})\Delta \delta_{x}(x_{c},y_{c})+a_{2}(x_{c},y_{c}){\left[\Delta \delta_{x}(x_{c},y_{c})\right]}^{2}\\ & +a_{3}(x_{c},y_{c}){\left[\Delta \delta_{x}(x_{c},y_{c})\right]}^{3}, \end{array}$$

where *a*_0_($x_{c}$, $y_{c}$), $a_{1}$($x_{c}$, $y_{c}$), $a_{2}$($x_{c}$, $y_{c}$), and $a_{3}$($x_{c}$, $y_{c}$) are the mapping coefficients of the cubic curve fitting.

**Figure 3:**
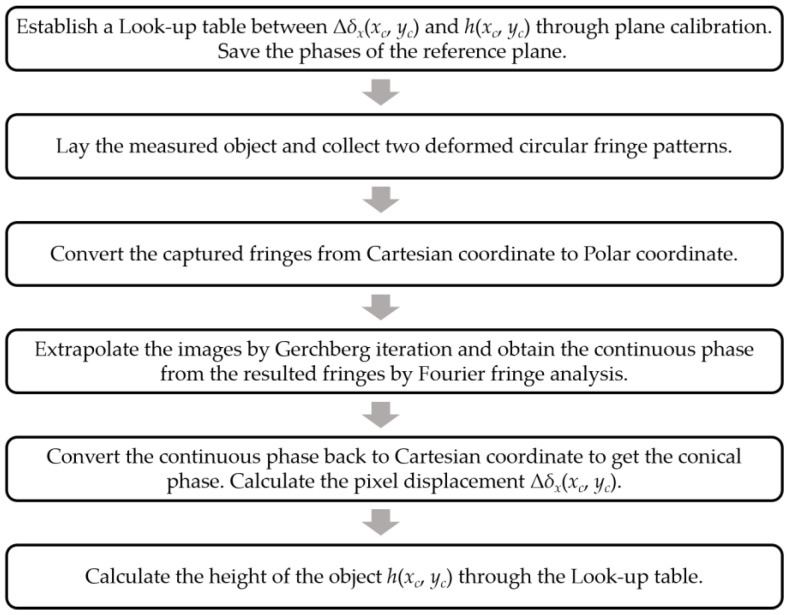
Flowchart of the improved CFFTP.

As two reference phases have been calculated and saved during system calibration, our method only needs to capture two deformed circular fringes to reconstruct the height of the measured object. A brief flow of the improved CFFTP is shown in Figure 3.

## 3. Simulations

**Figure 4:**
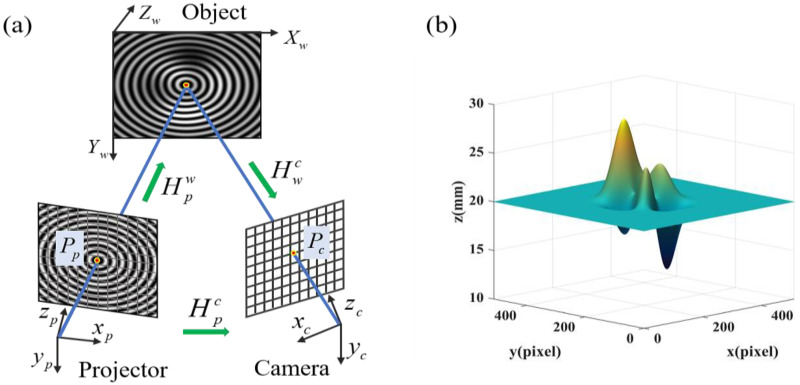
(**a**) Mathematical model of the virtual fringe projection system; (**b**)The simulated object when *H*(*x*, *y*) = 20 mm.

To verify the performance of our method, computer simulations are carried out for a telecentric system and non-telecentric system. For non-telecentric system simulation, a virtual fringe projection system based on the pinhole model is adopted for displaying the projection and imaging procedure, as shown in Figure 4a, where the subscript *w* denotes the world co-ordinate system, *c* denotes the camera image co-ordinate system and *p* denotes the projector co-ordinate system. The relationship between the camera co-ordinate system and the projector co-ordinate system can be rewritten as:

(18) $$P_{c}=H_{p}^{c}P_{p},$$

where $P_{c}$ and $P_{p}$ denote the corresponding homogeneous pixel co-ordinates in the camera co-ordinate system and the projector co-ordinate system, respectively. $H_{p}^{c}=H_{p}^{w}H_{w}^{c}$ is the homography matrix from the projector pixel co-ordinate system to the camera pixel co-ordinate system, where $H_{p}^{w}$ and $H_{w}^{c}$ represent the homography matrix from the projector pixel co-ordinate system to the world co-ordinate system and the world co-ordinate system to the camera pixel co-ordinate system, respectively. The detailed description was given in reference [34]. For a telecentric system, it is easy to generate the reference fringe and the deformed fringe by Equations (3)–(6) when $\delta_{x0}$ = 0.

First, assuming a telecentric system, a measured object mounted on a moving plane is simulated. The object *z*(*x*, *y*) is given as a peaks function multiplied by *S*, which can be expressed as:

(19) $$\begin{array}{ll} z(x,y)= & S\{3{\left(1-x\right)}^{2}\exp[-x^{2}-{\left(y+1\right)}^{2}]-10(\frac{x}{5}-x^{3}-y^{5})\exp(-x^{2}-y^{2})\\ & -\frac{1}{3}\exp[-{\left(x+1\right)}^{2}-y^{2}]\}+H(x,y), \end{array}$$

where *S* is a scalar factor, and *H*(*x*, *y*) denotes the corresponding height of the moving plane. In the simulation, we set *S* = 2, *H*(*x*, *y*) = 20, 30, 40 mm, respectively. Figure 4b shows the height distribution of the case of *H*(*x*, *y*) = 20 mm. The simulated radial period of fringes is 8 pixels, the lateral shift amount of circular centers of two reference fringes is 50 pixels, and the size of the fringe patterns is 512 × 512 pixels. Without loss of generality, Gaussian noise with 40 signal-to-noise ratio (SNR) is added to the simulated fringes.

**Figure 5:**
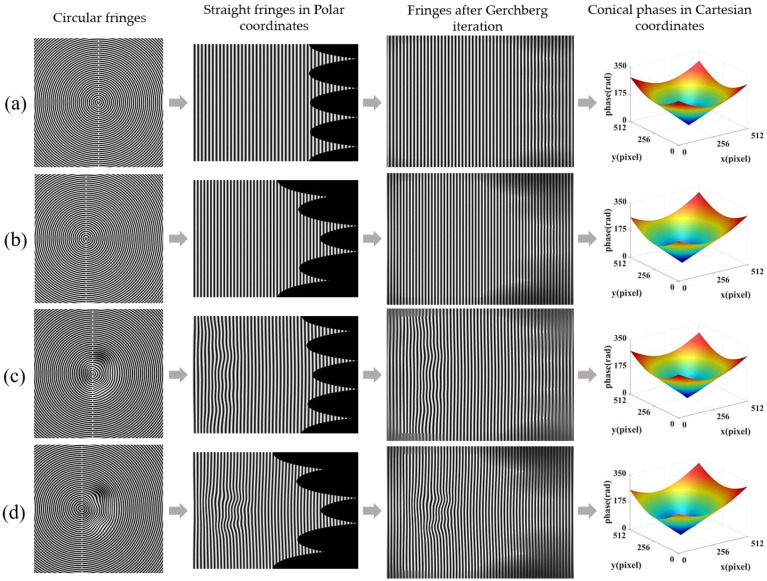
Simulated phase calculation process of improved CFFTP. (**a**) Conical phase calculation from reference fringe without lateral shift; (**b**) Conical phase calculation from reference fringe with lateral shift; (**c**) Conical phase calculation from deformed fringe corresponding to (**a**); (**d**) Conical phase calculation from deformed fringe corresponding to (**b**).

Figure 5 is the procedure of the conical phase calculation when *H*(*x*, *y*) = 20 mm. The first column of Figure 5a–d shows two reference circular fringe patterns and two deformed fringe patterns, respectively. The second column shows the corresponding linear fringes in the Polar co-ordinate. The third column shows the resulting fringes after Gerchberg iteration. The Fourier transform method is carried out on these extrapolated Polar co-ordinate fringes to calculate the corresponding wrapped phases. A spatial phase unwrapping algorithm [36] is utilized to obtain the continuous phase maps. Cutting the extrapolated areas, the phase maps of the four original fringes can be obtained. Then the phase maps are converted back to the original Cartesian co-ordinate system, as shown in the last column of Figure 5.

**Figure 6:**
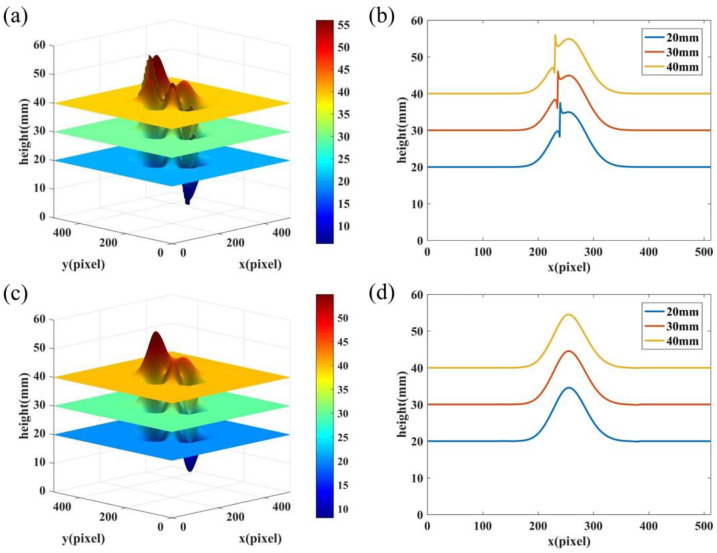
Reconstructed results by existing CFFTP and improved CFFTP when *H*(*x*, *y*) = 20 mm, 30 mm, and 40 mm, respectively. (**a**,**b**) Reconstructed surfaces and cross sections along the 330th row using existing CFFTP; (**c**,**d**) Reconstructed surfaces and cross sections along the 330th row using improved CFFTP.

Figure 6 shows the reconstruction of our improved CFFTP and that of the existing CFFTP. Figure 6a,b show the reconstructed 3D results and the cross sections along the 330th row by using the existing CFFTP. The corresponding results by using the improved CFFTP are shown in Figure 6c,d. It can be seen that both methods can correctly reconstruct the height range because the 2π ambiguity of unwrapping phases is avoided. However, there are obvious errors in the middle area by the existing CFFTP without fitting operation, while errors are eliminated by the improved CFFTP. To quantitatively evaluate the accuracy of the two methods, the root-mean-square error (RMSE) of reconstructions when *H*(*x*, *y*) = 20 mm, 30 mm, and 40 mm are exhibited in Table 1. The values of RMSE demonstrate that the proposed method provides much higher accuracy compared with the existing CFFTP.

**Figure 7:**
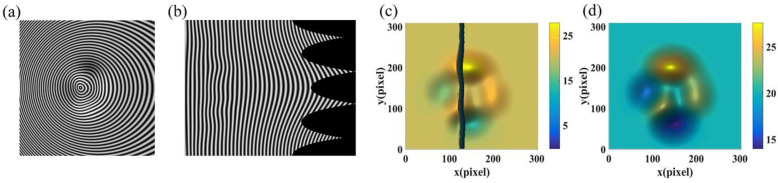
Simulation of non-telecentric system. (**a**) Deformed circular fringe; (**b**) Corresponding linear fringes in the Polar co-ordinate; (**c**) Reconstructed surface by existing CFFTP; (**d**) Reconstructed surface by improved CFFTP.

**Figure 8:**
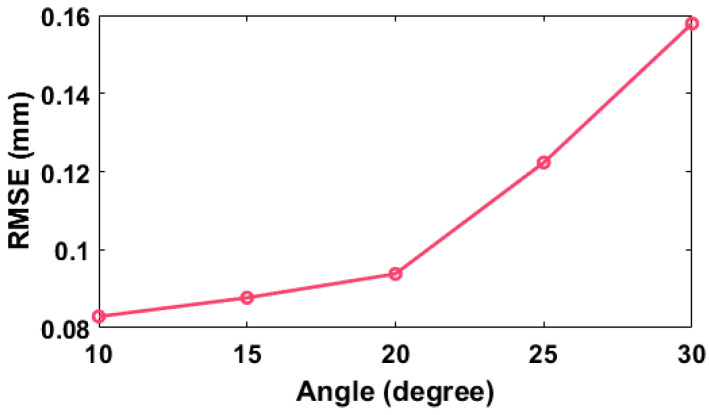
The RMSE distributions at different angles with improved CFFTP.

In the non-telecentric system, the period of the captured reference fringe is no longer a constant value. The larger the included angle of the optical axis, the bigger the period change of the reference fringe. To analyze the effect of the change of fringe period on reconstruction accuracy, the object in Figure 4b was measured multiple times by setting the angle of the optical axes at values of 10, 15, 20, 25, and 30 degrees, respectively. Figure 7 shows the simulation at the included angle of 20 degrees. Figure 7a is one of the deformed circular fringes. Compared with the circular fringe in Figure 5c, its period has changed due to the triangular relationship even in the region outside of the object. Figure 7b is its corresponding linear fringe in the Polar co-ordinate. Figure 7c,d show the enlarged reconstructed surface by the existing CFFTP and that by the improved CFFTP, respectively. The error in the middle area of the existing CFFTP is large without the fitting operation. In contrast, our method still can restore the object completely when the average period of the fringe is used to calculate Δ$\delta_{x}$. The RMSE distributions of the reconstructed result by our method at different included angles are shown in Figure 8. A bigger angle will cause a bigger error. Therefore, in the experiment, the included angle between the camera optical axis and the projector optical axis is set at an appropriate value (10–20 degrees).

It is noted that, in CFFTP, the period of the encoded fringe should be set to avoid frequency overlapping, like the traditional FTP. We find that as long as the fringe frequency is high enough to separate the fundamental component from other frequency components, the reconstruction accuracy will not be significantly affected when *p_c_* is approximated to the average value of the period of the captured reference fringe. A reasonable explanation is that the error caused by the period is regarded as a systematic error, which can be eliminated to a certain extent by the plane calibration.

## 4. Experiments

**Figure 9:**
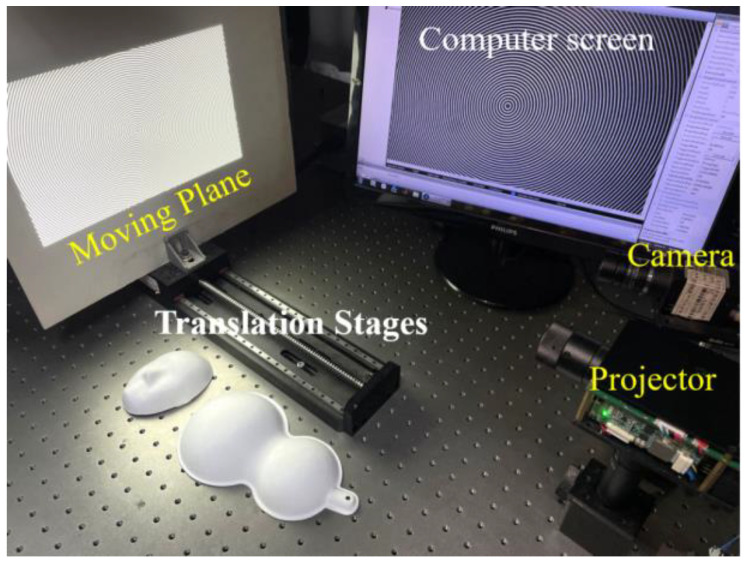
Experimental setup.

To further test the performance of the proposed method, we developed a measuring system and conducted a series of experiments. The experimental setup, shown in Figure 9, was mainly composed of a CCD camera (model: Vieworks VQ-5MG-M16) which uses an imaging lens with a focal length of 12 mm, a digital light processing projector (model: LightCrafter 4500), a flat white board (as reference plane) located on a translation stage (model: GCD-203300, repositioning precision is less than 5 μm), and a computer for controlling and calculating. The resolutions of the camera and the projector are, respectively, 2448 × 2048 pixels and 912 × 1120 pixels. The angle between the camera and the projector was set to 15 degrees approximately. In the following experiments, two circular fringe patterns with a radial period of 10 pixels were generated, and the lateral shift amount of their circular centers was 50 pixels.

Before the measurement, the plane calibration was performed to establish the displacement-to-height mapping table. During the calibration procedure, the reference plane mounted on the translation stage was moved from 0 to 60 mm with 10 mm as an interval. At each position, the lateral displacement of the whole image was calculated according to Equation (9). Then the coefficients of Equation (17) were estimated to make a look-up table and stored in the computer.

**Figure 10:**
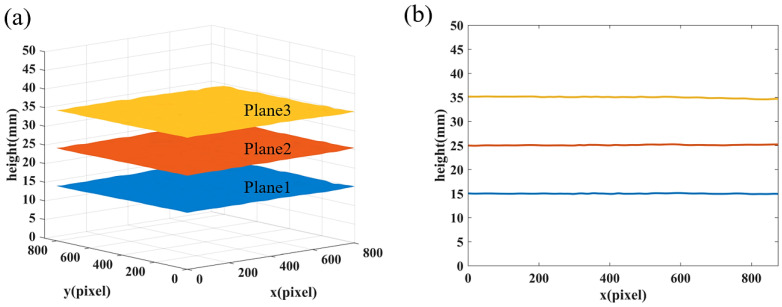
Experimental results of the planes at different heights. (**a**) 3D reconstruction; (**b**) Corresponding cross section.

In the first experiment, three out-of-plane heights located at the positions of 15.0000 mm, 25.0000 mm, and 35.0000 mm were respectively measured to examine the precision of the calibration. The measurement results and RMSE for each flat plane are listed in Table 2. Figure 10a shows the 3D reconstruction results of three planes, and Figure 10b displays the cross sections of the results.

It can be seen that our method can restore the height of planes correctly since the unwrapping phases have no 2π ambiguity. However, the measurement error of CFFTP might be bigger than that of linear fringe projection FTP with marker points because this method includes additional co-ordinate transformation operations.

**Figure 11:**
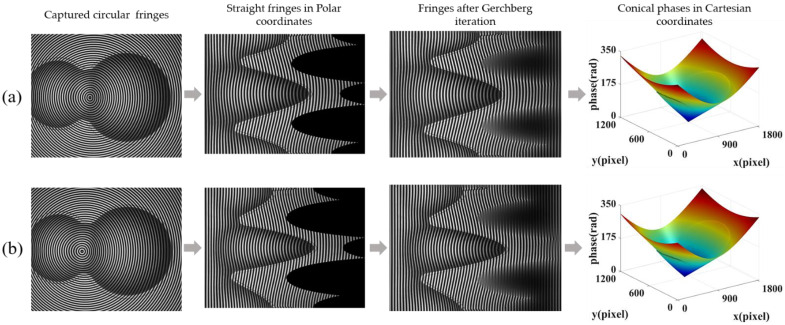
(**a**) Conical phase calculation from the captured fringe without lateral shift; (**b**) Conical phase calculation from the captured fringe with lateral shift.

**Figure 12:**
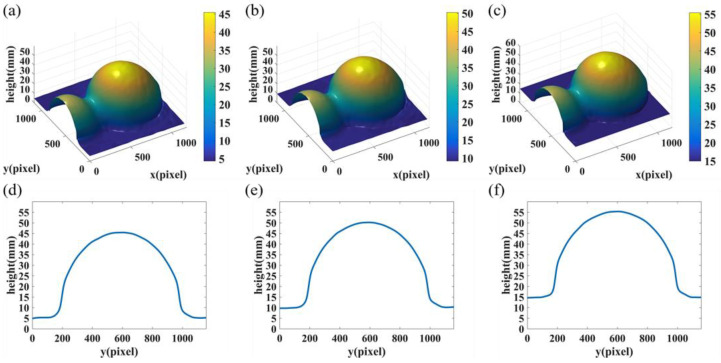
Reconstructed results of the gourd. (**a**–**c**) 3D reconstruction at the position of 5mm, 10mm, and 15mm, respectively; (**d**–**f**) Cross sections of (**a**–**c**) along the 720th column.

To further demonstrate the validity of the proposed method in out-of-plane measurement, a gourd-shaped object mounted on a moving plane was measured. We chose three positions with heights of 5.0000 mm, 10.0000 mm, and 15.0000 mm as the measurement samples. Taking the height of 5 mm as an example, Figure 11a,b display the process of obtaining the conical phase from two captured fringes without and with lateral shift, respectively. From left to right, the captured fringes, the resulting fringes in the Polar co-ordinate system before and after Gerchberg iteration, as well as the final conical phases are shown. To eliminate the non-uniform background intensity caused by illumination and reflection, FTP with background homogenization was used to process these fringes [37]. The reconstructed height of the object at different positions are shown in Figure 12a–c, respectively, where color bars display the range of the reconstruction results in color. Figure 12d–f are the cross sections along the 720th column of the 3D shapes shown in Figure 12a–c.

**Figure 13:**
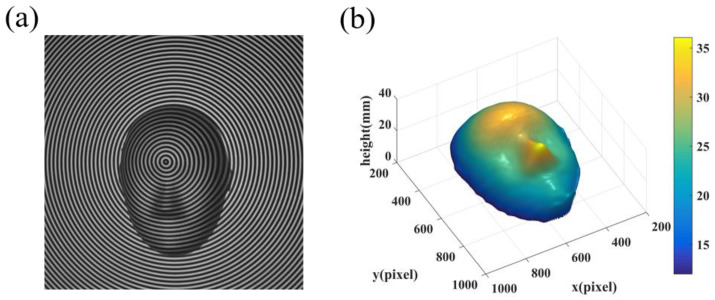
Reconstructed results of the mask. (**a**) Captured deformed fringe pattern; (**b**) 3D reconstruction.

In another experiment, a mask was measured by our method. The mask was placed on the reference plane which was moved forward 12.0000 mm. The captured deformed fringe is shown in Figure 13a, and the 3D shape of the object is shown in Figure 13b. These measurements show that the proposed CFFTP can reconstruct the 3D shape of the out-of-plane object correctly.

## 5. Conclusions

In this paper, an improved CFFTP method has been proposed for out-of-plane measurement with higher accuracy. By projecting an additional circular fringe pattern with a center shift, the method retrieves the pixel displacement from a linear equation instead of a quadratic equation. Therefore, the calculation process is simpler and more reliable compared with the existing CFFTP. Subsequently, the influence of non-uniform period in non-telecentric triangulation system is discussed in the theoretical analysis. The plane calibration method is used to create a look-up table between displacement and height to eliminate the systematic error of the improved CFFTP. In addition, Gerchberg iteration is employed to eliminate phase error in the middle region. The simulation results and experiments on different objects demonstrate the validity and feasibility of the proposed method. We will further explore error compensation methods to improve accuracy, such as eliminating the error caused by the non-uniform reference fringe period in a non-telecentric system and insufficient sampling rate in some areas during the co-ordinate transformation operation.

## Figures and Tables

**Table 1:** RMSE of the reconstructed object at three positions by two methods. (Unit: mm).

Method	Position		
	20	30	40
Existing CFFTP	0.1875	0.2038	0.2198
Improved CFFTP	0.0623	0.0746	0.0874

**Table 2:** Measurement results for the out-of-plane height. (Unit: mm).

Height	Mean	RMSE
15.0000	14.9683	0.1183
25.0000	25.0570	0.1227
35.0000	35.0328	0.1443

## Data Availability

Not applicable.
